# Change in Functional Moderate Mitral Regurgitation after Aortic Valve Replacement

**DOI:** 10.21470/1678-9741-2018-0331

**Published:** 2019

**Authors:** Weitie Wang, Tiance Wang, Hulin Piao, Bo Li, Yong Wang, Dan Li, Zhicheng Zhu, Rihao Xu, Kexiang Liu

**Affiliations:** 1 Department of Cardiovascular Surgery, Second Hospital of Bethune, Jilin University, Changchun, Jilin, People’s Republic of China.

**Keywords:** Mitral Valve Insufficiency, Ventricular Remodeling, Atrial Fibrillation, Stroke Volume, Aortic Valve Stenosis, Echocardiography, Retrospective Studies

## Abstract

**Objective:**

To evaluate the changes of the mitral valve geometrics and the degrees of moderate mitral regurgitation (MR) in patients undergoing aortic valve replacement (AVR) for aortic stenosis (AS).

**Methods:**

A retrospective analysis study of intraoperative transesophageal echocardiography (TEE) and postoperative transthoracic echocardiography (TTE) was performed in 49 patients diagnosed with pure AS combined with moderate MR, who underwent AVR from January 2013 to December 2017. TEE was used to evaluate the direct geometric changes of the mechanical effects on mitral annulus after AVR. TTE was used to evaluate the changes of MR after operation. All patients underwent TTE during the midterm follow-up. The mean follow-up time was 40.21 months.

**Results:**

All of the 49 patients had moderate MR. Anterolateral-posteromedial diameter, anterior-posterior diameter, and mitral annular area were significantly reduced after AVR, while no significant changes were found in the intraoperative left ventricular loading conditions before and after AVR. The degree of mitral valve regurgitation, left ventricular size, left atrial size, left ventricular end-diastolic volume, and left ventricular to aortic pressure gradient were significantly reduced before discharge, and midterm follow-up showed good results.

**Conclusion:**

This study supports the belief that aortic outflow tract obstruction and an actual mechanical compression of the anterior mitral annulus after AVR would cause reduction in MR. Ventricular remodeling would also cause reduction in MR with time going on. Patients with AS, especially young patients with moderate MR, were most likely to benefit from AVR in early time.

**Table:** 

Abbreviations, acronyms & symbols				
AF	= Atrial fibrillation		IABP	= Intra-aortic balloon pump
AL-PM	= Anterolateral-posteromedial		ICU	= Intensive care unit
AP	= Anterior-posterior		LA	= Left atrial
AS	= Aortic stenosis		LCOS	= Low cardiac output syndrome
AVR	= Aortic valve replacement		LV	= Left ventricular
BMI	= Body mass index		LVEDV	= Left ventricular end-diastolic volume
COPD	= Chronic obstructive pulmonary disease		LVEF	= Left ventricular ejection fraction
CPB	= Cardiopulmonary bypass		MR	= Mitral regurgitation
CRD	= Chronic renal dysfunction		NYHA	= New York Heart Association
CVP	= Central venous pressure		SD	= Standard deviation
DM	= Diabetes mellitus		SPSS	= Statistical Package for the Social Sciences
EDV	= End-diastolic volume		TEE	= Transesophageal echocardiography
EF	= Ejection fraction		TTE	= Transthoracic echocardiography

## INTRODUCTION

Left ventricular (LV) remodeling and increased afterload caused by aortic outflow tract obstruction might lead to mitral regurgitation (MR)^[1]^. It is a functional MR, commonly coexisting with aortic stenosis (AS). Surgical intervention must be carefully selected because double valve surgery is more associated with increased mortality^[2]^ than isolated aortic valve replacement (AVR). Besides, additional mitral valve intervention during AVR had been long debated, especially for functional MR^[1-4]^. It was important to improve the degree of moderate MR after AVR. Up to now, some studies with small patient cohort and short-term follow-up results had been reported on this topic^[3,4]^, but the results remained controversial. In addition, the cases in most of the published reports were patients with relatively old ages. So, our study aimed to examine the direct influence and following changes on mitral valve after AVR in young patients based on a single-center experience.

## METHODS

This study was conducted as a retrospective observation from January 2013 to December 2017 patients and was approved by the Ethics Committee of the Jilin University. All surgeries were performed by the same surgeon. A total of 1,100 patients were admitted into the Department of Cardiovascular Surgery of the Second Hospital of Bethune, Jilin University, for surgical valve treatment during this period. Data of transesophageal echocardiography (TEE) and postoperative transthoracic echocardiography (TTE) were collected and analyzed. All patients were diagnosed with pure AS coexistent with moderate MR (MR+2-3) by two-dimensional echo examination TTE. All patients underwent isolated AVR and patients with other cardiac diseases requiring concomitant management at that time were excluded. Finally, 49 patients were included in this study.

Echocardiographic examinations were performed using a Philips IE33 system (Philips Medical System, Andover, Massachusetts). Intraoperative TEE was used to evaluate the direct geometric changes on the mitral annulus after AVR. TEE was used in the intraoperative periods, before and after cardiopulmonary bypass (CPB). TTE was used to compare the differences between preoperative and post-discharge parameters. The etiology of MR was determined by the echocardiogram, and organic abnormalities, such as ischemic, rheumatic, or degenerative etiologies associated with evidence of leaflet, annular, chordal, or papillary muscle pathology, were excluded. In addition, patients with coronary artery diseases were also excluded from the present study. Coronary angiography was arranged if the man was over 50 years old or the woman was over 55 years old.

All data, including preoperative, intraoperative, and postoperative information, were collected from our echocardiographic database. Baseline clinical data included age, sex, obesity, smoking, New York Heart Association (NYHA) class, diabetes mellitus (DM), chronic renal dysfunction (CRD), preoperative atrial fibrillation (AF), congestive heart failure, hypertension, hyperlipidemia, chronic obstructive pulmonary disease (COPD), prior cerebrovascular accidents, and left ventricular ejection fraction (LVEF). Operative data, including operation time, cross-clamp time, CPB time, and valve type, were collected. Postoperative outcomes (survival or death) included length of intensive care unit (ICU) and hospital stays, atrial and ventricular arrhythmia, and major procedure-related complications: respiratory complications, neurologic complications (stroke or transient ischemic attack), and low cardiac output syndrome (LCOS). Intraoperative TEE findings included anterolateral-posteromedial (AL-PM) diameter, anterior-posterior (AP) diameter, mitral annular area, left ventricular end-diastolic volume (LVEDV), and left atrial (LA) and LV sizes. Echocardiographic findings included LVEF, LVEDV, grade of MR, and LA and LV sizes.

### Grading of Mitral Regurgitation

MR grading was established according to the European Society of Echocardiography diagnostic criteria^[5]^. It was strictly quantified 0 (none), 0.5 (trace), 1 (mild), 1.5 (mild-moderate), 2 (moderate), 3 (moderate-severe), and 4 (severe). Moderate MR was defined as grades 2 or 3 of regurgitation.

TEE was employed during operation to re-examine the valve situation and to evaluate the surgical effects of AVR and other parameters such as annular area, AL-PM diameter, and AP diameter. The echocardiogram was analyzed by two experienced associate professors who were specialized in cardiac ultrasonography.

The primary end points were the degree of MR before and after surgery and the changes in mitral annular area. The secondary end points were overall death, including in-hospital mortality and death after discharge, as well as the occurrence of major postoperative morbidities, such as LCOS, perivalvular leak, and other cardiac-related complications. Follow-up information was obtained by outpatient visit or telephone calls and was agreed by all patients before discharge with informed consent. All surviving patients underwent postoperative echocardiographic re-examinations. The mean follow-up time was 40.21 months.

### Definitions

Surgical mortality was defined as death occurring in hospitalization and/or within 30 days of the procedure. The secondary end points were other complications related to the operation. Resternotomy for bleeding was defined as reoperation to control bleeding within 36 hours following the initial surgery. Postoperative LCOS was defined as the requirement for intra-aortic balloon pump (IABP) and/or inotropic support for longer than 30 min. Atrial/ventricular arrhythmia was any episode of atrial/ventricular fibrillation that was identified by the monitoring system on a rhythm strip or the 12-lead electrocardiogram. Postoperative respiratory failure was defined as duration of mechanical ventilation more than 72 hours or reintubation following surgery. Postoperative pneumonia was a positive result in a sputum culture requiring anti-infective treatment or a chest X-ray diagnosis of pneumonia following cardiac surgery. Stroke was new onset of permanent neurological event lasting over 24 hours. Deep sternal wound infection was bone related, including any drainage of purulent material from the sternotomy wound and instability of the sternum.

### Statistics

All statistical analyses were performed by the Statistical Package for the Social Sciences (SPSS) software, version 19.0. Continuous data were expressed as a mean±standard deviation (SD). Normally and non-normally distributed continuous variables were compared using Student’s t-test and Mann-Whitney U test, respectively. Categorical variables were compared using Chi-square tests. SNK-q test was used for multiple comparisons among groups. Values of *P*<0.05 were considered statistically significant.

## RESULTS

### Study Population

There were 49 patients in this study. These patients were predominantly young, with an age range of 52.61±12.23 years. All patients underwent AVR only. More detailed baseline characteristics were shown in Table 1, including age, gender, obesity, smoking, NYHA class III-IV, hypertension, DM, CRD, congestive heart failure, hyperlipemia, COPD, prior cerebrovascular accident, and LVEF.

**Table 1:** Baseline and procedural characteristics.

	AS with moderate MR (n=49)
Age (years)	52.6±12.2
Age > 60 years	9(18.4%)
Males	22(44.9%)
Obesity (BMI > 30 kg/m^2^)	2(4.5%)
Smoking	21(42.9%)
NYHA class III-IV	35(71.4%)
Hypertension	19(38.8%)
Diabetes mellitus	11(22.5%)
Chronic renal dysfunction	0
Congestive heart failure	4(8.2%)
Atrial fibrillation	0
Hyperlipemia	5(10.2%)
COPD	5(10.2%)
Prior cerebrovascular accident	2(4.5%)
LVEF	56.8±12.9%
LVEF < 50%	8(16.3)
Bicuspid aortic valve stenosis	18(36.7%)
Rheumatic disease	13(26.5%)
Calcific disease	18(36.7%)

AS=aortic stenosis; BMI=body mass index; COPD=chronic obstructive pulmonary disease; LVEF=left ventricular ejection fraction; MR=mitral regurgitation; NYHA=New York Heart Association

### Intraoperative Outcomes

Intraoperative data were shown in Table 2. CPB time was 32.5±7.3 min and cross-clamp time was 24.3±3.4 min. Prosthetic valves included 37 mechanical prostheses and 12 tissue valves (St. Jude Medical Epic valves, size 17-23 mm; St. Jude Medical Inc., St. Paul, MN, USA).

**Table 2:** Intraoperative data.

	AS with moderate MR (n=49)
Operation time (min)	208.2±31.2
Cardiopulmonary bypass time (min)	32.5±7.3
Cross-clamp time (min)	24.3±3.4
Mechanical valves	37
Tissue valves	12

AS=aortic stenosis; MR=mitral regurgitation

### Postoperative Outcomes

Chest tube drainage during the first 12 hours was 259.2±67.1 ml. Time of mechanic ventilation was 7.5±2.2 hours. Length of ICU and hospital stays were 2.0±0.2 days and 10.4±0.2 days, respectively. No severe postoperative complications were found. More details were shown in Table 3.

**Table 3:** Postoperative data.

	AS with moderate MR (n=49)
Surgical mortality	0
Resternotomy for bleeding	0
Duration of mechanic ventilation (hour)	7.5±2.2
Intensive care unit stay (days)	2.0±0.2
Hospital stay (days)	10.4±0.2
Ventricular arrhythmia	0
Atrial arrhythmia	6(12.2%)
Respiratory failure	0
Pneumonia	0
Neurologic complications	0
Low output syndrome	0
Drainage during the first 12 hours (ml)	259.2±67.1
Tamponade	0
Stroke	0
Deep sternal wound infection	0

AS=aortic stenosis; MR=mitral regurgitation

### Change in MR During Operation

The mitral valve geometry changed significantly after AVR with the annular area decreasing by 17.7%, reducing from 5.4±0.5 to 4.5±0.2 cm^2^ (*P*<0.001). The AL-PM diameter decreased by 14.0%, from 3.7±0.2 to 3.2±0.2 cm (*P*<0.001), and the AP diameter decreased by 7.1%, from 1.8±0.1 to 1.1±0.1 cm (*P*<0.001). No significant change was seen in the LVEDV (125.56±16.4 *vs*. 124.7±15.9 ml, *P*=0.7833), LV size (39.8±2.3 *vs*. 39.7±2.4 mm, *P*=0.8337), and LA size (32.4±2.3 *vs*. 31.9±2.2 mm, *P*=0.2742). More details were shown in Table 4.

**Table 4:** Intraoperative transesophageal echocardiographic data.

Variable (n=49)	Pre-CPB (n=49)	Separation from CPB (n=49)	*P*-value
Annular area (cm^2^)	5.4±0.5	4.4±0.2	<0.001
AL-PM diameter (cm)	3.7±0.2	3.2±0.2	<0.001
AP diameter (cm)	1.8±0.1	1.7±0.1	<0.001
LVEDV (ml)	125.6±16.4	124.7±15.9	0.7833
LV size (mm)	39.8±2.3	39.7±2.4	0.8337
LA size (mm)	32.4±2.3	31.9±2.2	0.2742

AL-PM=anterolateral-posteromedial; AP=anterior-posterior; CPB=cardiopulmonary bypass; LA=left atrial; LV=left ventricular; LVEDV=left ventricular end-diastolic volume

### Postoperative Outcomes of MR

**Fig. 1:**
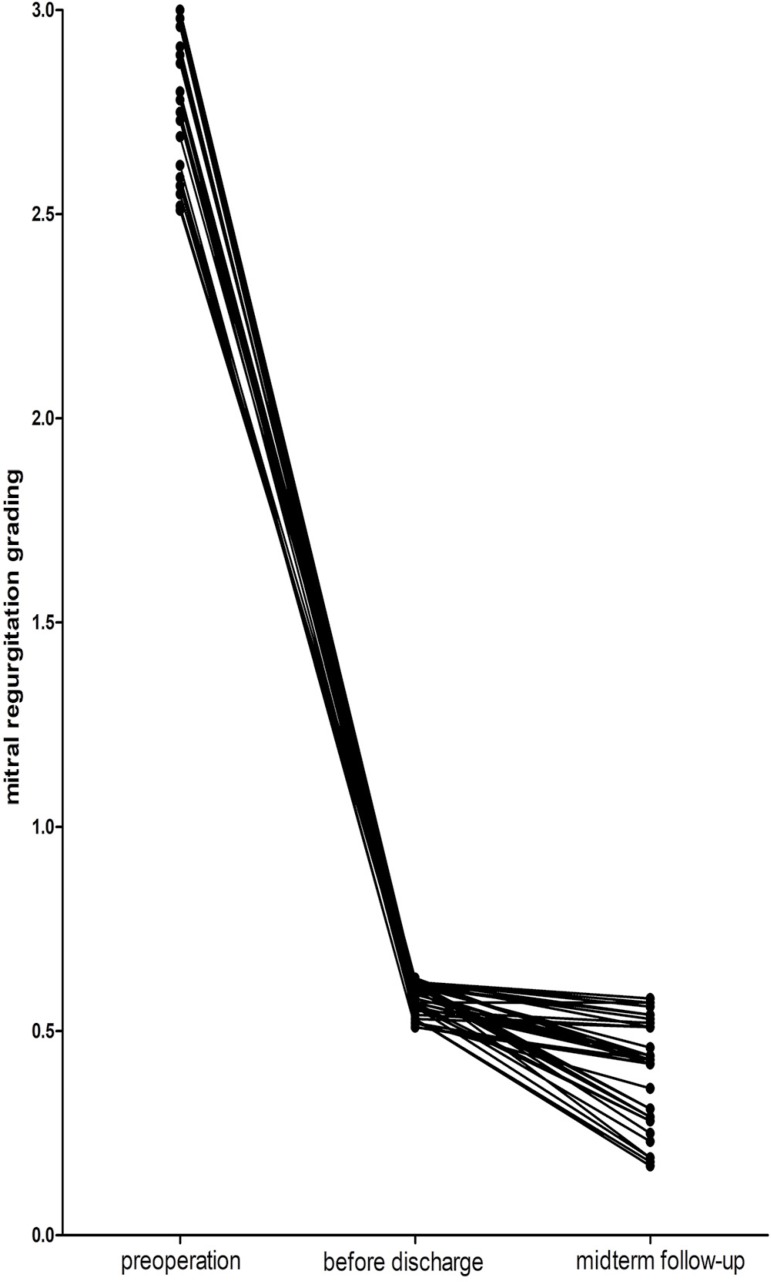
Changes in grade of mitral regurgitation after aortic valve replacement.

All patients survived in hospital and were discharged with no symptom caused by remaining MR. All patients had a 40.21 (8-60) months follow-up. MR grade was significantly decreased from 2.9±0.2 to 0.6±0.04, and during the midterm follow-up, MR grade was 0.4±0.1, which was different from 10 days after operation (*P*<0.05). The re-examination during follow-up (Figure 1) showed that trace grade was present in 38 (77.55%) patients and trace-mild grade was present in 11 (22.45%) patients (χ^2^=98.00, *P*<0.05).

The LA size significantly changed from 37.4±2.2 mm to 30.7±2.0 mm and decreased to 27.4±1.5 mm during midterm follow-up, which presented a significant difference between preoperative and postoperative periods (*P*<0.05). Statistical difference of LV reversal remodeling was observed through the size of LV (53.8±2.1 mm vs. 41.2±3.8 mm, *P*<0.05) and the LVEDV (146.8±15.3 ml *vs*. 95.3±6.2 ml, *P*<0.05) at 10 days after operation comparing with preoperative data. As time went by, size of LV (41.0±3.4 mm) and LVEDV (94.1±7.1 mm) decreased, but had no significant difference with 10 days after operation (*P*<0.05). LVEF was lower (51.7±6.3%) in the postoperative period (10 days) than in the preoperative period (53.7±8.4%), but it improved significantly after midterm follow-up (57.0±7.0%), comparing with the preoperative period. Mean NYHA class improved during follow-up (3.3±0.3 *vs*. 2.0±0.1). LV to aortic pressure gradient dropped significantly from 117.4±26.2 mmHg to 32.1±14.4 mmHg, and continuously decreased to 27.2±8.1 mmHg, which had significant difference from preoperative period and 10 days after operation (*P*<0.05) (Table 5).

**Table 5:** Transthoracic echocardiographic data.

Variable	Before operation	10 days after operation	Midterm follow-up after operation	*F* or Χ^2^
Patients	N=49	N=49	N=49	
LA size	37.4±2.2	30.7±2.0^a^	27.4±1.5^ab^	344.1*
LV size	53.8±2.1	41.2±3.8^a^	41.0±3.4^a^	259.9*
EDV	146.8±15.3	95.3±6.2^a^	94.1±7.1^a^	412.0*
EF	53.7±8.4	51.7±6.3^a^	57.0±7.0^ab^	6.612*
MR	2.9±0.2	0.6±0.04^a^	0.4±0.1^ab^	5498*
Mean NYHA class	3.3±0.3	2.0±0.1^a^	1.5±0.1^ab^	1154*
Left ventricular to aortic pressure gradient	117.4±26.2	32.1±14.4^a^	27.2±8.1^ab^	394.2*

* *P*<0.05

a = compare with before operation data;

b = compare with 10 days after operation data EDV=end-diastolic volume; EF=ejection fraction; LA=left atrial; LV=left ventricular; MR=mitral regurgitation; NYHA=New York Heart Association

## DISCUSSION

AS has become the most prevalent disorder of all valvular heart diseases^[6]^. It is more frequent as the age increases, due to calcification. However, in young patients, bicuspid valve became another main etiology of AS and the stenosis in bicuspid valve patients arises nearly two decades before than those in tricuspid aortic valves. But in some rural areas, rheumatic valve disease is still a main cause of AS^[7]^. A slight predominance of this disorder was noted in male patients. But in this study, the age of male patients who need coronary angiography was lower than of female patients. So, more male patients with different degrees of coronary disease were excluded than females, which might result in the unusual situation of female preponderance in this patient population.

Mild to moderate MR was always found in patients with AS. It was a functional disease caused by the increase of transaortic pressure^[3]^ and LV remodeling. So, after AVR, decrease of transaortic pressure and LV remodeling by the reduced afterload might reduce the mitral annular size and functional MR grade. Therefore, some experts suggested that the functional MR did not require interventions because they found out that 95% of AS patients with moderate and mild to moderate MR would decrease at least one degree of MR after AVR^[8]^. Matsumura et al. showed that 36% of AS patients with moderate MR would not benefit from AVR. Kowalówka et al. showed that moderate MR remained unchanged in 19-38% and deteriorated in 1-14% of patients after AVR^[3,4]^. Actually, combined aortic and mitral valve replacement presented a high risk during the operation and affected the survival rate (in-hospital mortality, 15.5%; median survival rate, 7.3 years)^[9]^. In addition, Zilberszac et al.^[10]^ claimed that concomitant MR would not significantly improve the survival rate. However, Sorabella et al.^[11]^ showed that patients undergoing isolated AVR with concomitant 2-3+ MR experience poorer long-term survival than those patients with no or mild functional MR. Accordingly, surgical intervention, especially double valve replacement, must be carefully selected^[12]^.

A previous report^[13]^ claimed that the direct mechanical compression of the anterior mitral annulus played an important role in MR. Sehovic et al.^[14]^ also reported the same result and pointed out that 53.3% of moderate regurgitation decreased after AVR at an early time. This study suggested that the reduction in intraventricular pressure after AVR seemed to be the most important mechanism related to decreased MR. However, compared with the directly decreased ventricular systolic pressure and fluid overload, LV remodeling could also contribute to the decreased MR^[15]^, as the time went by. So, this study aimed to evaluate the direct effects of isolated AVR on moderate MR and to examine the determinants of postoperative changes in MR.

In our study, organic MR and patients with coronary artery diseases were also excluded because ischemic heart diseases could also cause functional MR^[16]^. Many reports showed that there were some predictors associated with the decrease of postoperative MR, such as LA size > 45 mm, pulmonary artery systolic pressure > 40 mmHg, and LVEDV > 54 mm^[14]^. In this study, only one case of LA > 45 mm was observed and he also recovered with decrease of MR after AVR. In addition, the LV size of female patients in the study was smaller than male one, but the large LV size in males also showed excellent results after AVR. Although the age of the patients was associated with improvement in MR, it was not found in our study. This may be because this study had younger patients than other studies and our strict criteria for surgery.

After AVR, MR severity was assessed by the regurgitant volume decreased in all patients. The mean reduction was 90% in the regurgitant volume, emphasizing the importance of the reduction in systolic LV pressure. As the pressure of LV cavity sharply dropped after AVR, the transmitral pressure also decreased, and they both contributed markedly to the decrease in the MR volume. So, these results suggested that systolic LV pressure reduction was a main contributor to the early postoperative reduction in regurgitant volume.

**Fig. 2:**
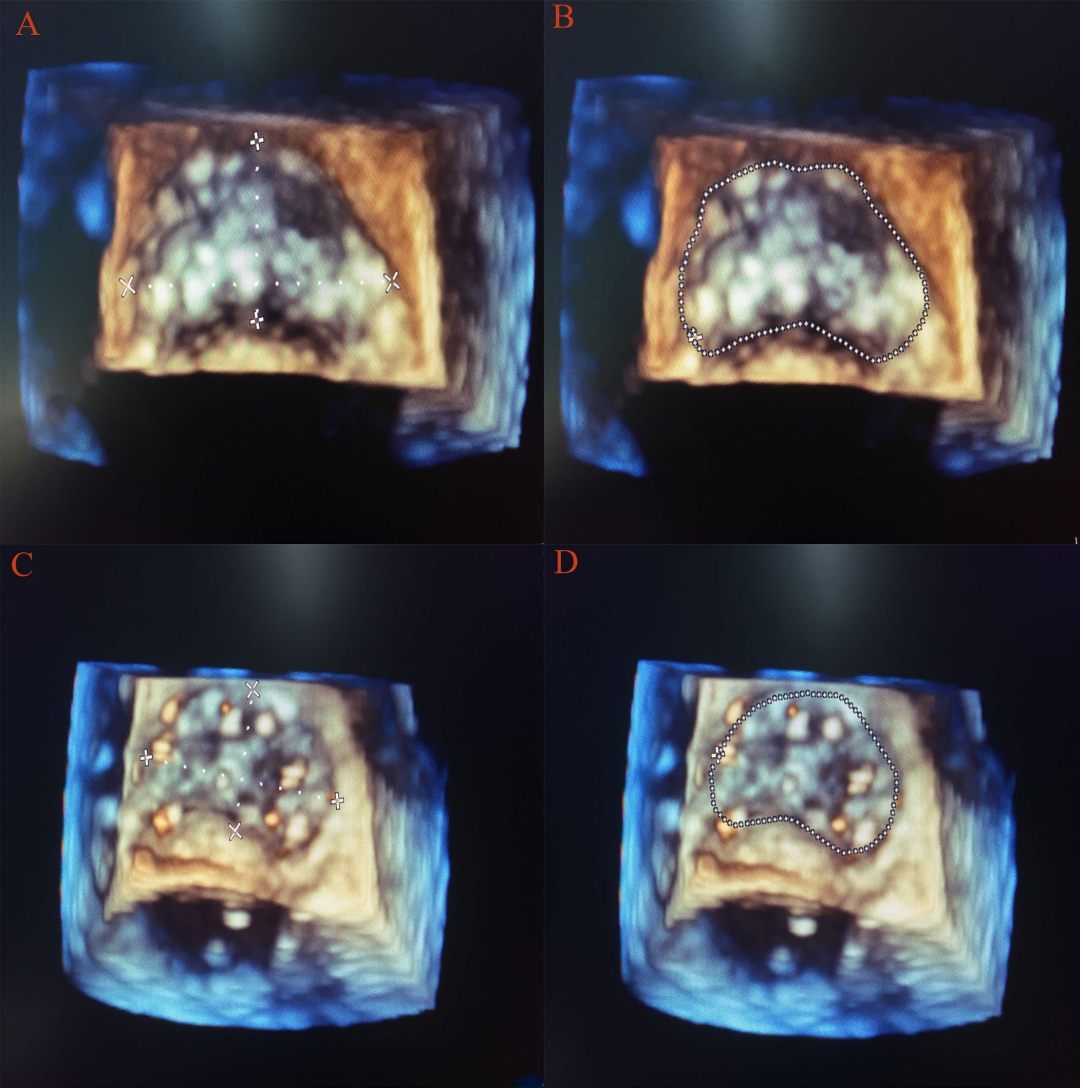
Changes in 3D mitral valve model after aortic valve replacement (AVR). Screen capture demonstrates a completed 3D mitral valve model. The 2D long-axis echocardiographic views represent orthogonal planes with a cross-sectional coronal plane for annular area tracing. The 3D model shows the final model with automatically labeled landmarks and diameters. A, B=Pre-AVR analysis. C, D=Post-AVR analysis in the same patient demonstrating a reduction in the mitral annular area (from 5.91 to 4.25 cm2), anterior-posterior diameter (from 1.92 to 1.68 cm), and anterolateral-posteromedial diameter (from 3.80 to 3.27 cm).

As we all know, the mitral and aortic valves were contiguous and shared the fibrous ‘aorto-mitral curtain’ (anterior mitral annulus with the aortic valve). So, after AVR, the mechanical effects of a rigid prosthesis in the aortic position would immediately cause geometric changes in the mitral annulus, which was another contributor to the early postoperative reduction in regurgitant volume. As assessed with real-time three-dimensional TEE, the mitral annular area was significantly reduced in our study. The reductions in mitral annular area after AVR were similar to what occurred with the implantation of a full annuloplasty ring^[17]^ in the mitral position to reduce the mitral annular area in order to prevent MR from progressive annular dilation. But the reduction in anterior and posterior annulus length cannot be measured because of lacking of the mitral valve assessment package, though it was obvious to see the change through 3D picture (Figure 2). At the same time, a statistically significant reduction in the AL-PM diameter after AVR in our study also strongly suggested that there might be an element of mechanical compression of the mitral annulus anteriorly over and above the acute reduction in afterload as a possible etiological factor. But the impact of acute unloading of the ventricle immediately after AVR cannot be excluded as a possible contributory mechanism. During evaluation of the change of such parameters, we tried to select a more stable period before and after CPB. In order to avoid bias, the evaluation started when the central venous pressure (CVP) and LVEDV after AVR were similar to pre-CPB values. Though acute reductions in afterload and transmitral pressure would act as a possible etiological factor to reduce the MR, mechanical compression of the aorto-mitral curtain was particularly significant as the reduction of AP annular dimension. After all, the AP annular dimension was still the most common axis of annular dilation/contraction with changes in loading conditions. Due to the fact that the loading conditions were similar during calculation, we believed that an actual mechanical compression of the aorto-mitral curtain might act as a possible cause of the reduction of MR that was consistent with the findings of a previous study^[13]^.

At the same time, we recognized that LV remodeling was not immediately observed in TEE during operation, as assessed by the LV size, which was nearly the same as the preoperative data. But after 10 days before discharge and in the midterm follow-up, it proved to be a favorable LV remodeling, due to a chronic reduction of afterload. Changes in LV shape and geometry might decrease in mitral tethering forces and finally improve MR grade. Such LV remodeling was probably the consequence of the correction of afterload. LV size decreased a lot since the preoperative period and made LV size seem small. Actually, the LV size was influenced by many factors, especially volume status. More stringent rules on controlling the input and output fluid after operation was another main influence factor on the LV size. In addition, the pattern of the LV hypertrophy was an adaptive response to pressure overload in AS. Concentric remodeling would increase wall thickness and decrease the size of LV chambers. In addition, the excellent results of our study were also more relevant with the small LV size after operation than with those with large LV size^[16]^.

Most of the patients who were classified as NYHA class III/IV seem to mismatch with the LV size in this study. It was a little hard to evaluate the patients’ NYHA class just on the complaint and activity endurance. In China, especially in some rural areas, most patients’ complaints were always influenced by many factors, such as their educational background and other patients’ symptoms, as well as their own thoughts. So, some of these 49 patients’ complaints and activity endurance were always very severe when they were in hospital, and they expected to get an earlier operation chance than those with less serious complaints and activity endurance. In addition, there were nine patients with EF < 50% in this study. After cardiotonics and diuretics, the symptom was improved in these patients. Whether the grade of MR of these nine patients after cardiotonics and diuretics treatment had changed or not was not sure. However, the grade of MR of the first echocardiography was not significantly different from those with relatively normal EF.

Our study showed excellent results on the reduction of MR, which might be due to the strict inclusion criteria. Any suspicion of organic mitral valve disease was an excludable factor from our study. In addition, more stringent applications on controlling the input and output after operation also played an important role in improving the MR. In this study, patients were treated with medicine before surgery, which had a good effect on decreasing MR grade after operation. The patients with EF < 50% got the maximized benefits from decreasing MR grade after correcting heart function. Last, references^[3,18]^ showed that 95% and 88% of young patients with moderate MR seem to get better results after AVR, which was consistent with our study.

### Limitation

The current trial has several limitations. Firstly, it is a retrospective observational study within a single center, which may influence the generalizability. So, a final determination would require a prospective, multi-center study with larger sample size than this. Secondly, latest changes in preload or afterload were associated with general anesthesia, which were additional limitations for interpreting this study, so the grade of MR after anesthesia was not compared. In addition, issues related to post CPB hyperkinesia, with the use of inotropics, can alter hemodynamic status and cardiac dynamics. Thirdly, little atrial regurgitation combined with AS was also included in patients which may produce biased results. Some young patients did not undertake coronary angiography and cannot totally be excluded from ischemic heart diseases. Finally, this essay has no control group because AVR plus mitral-valve repair was carried in recent years in our department, which only had 19 cases. So, a larger sample size than this should be added as a control group in the future.

## CONCLUSION

To conclude, our study showed that patients with moderate regurgitation were more likely to benefit from conservative AVR surgery only. The mechanical compression and relief of aortic outflow tract obstruction after AVR would directly cause the reduction in MR. Ventricular remodeling would also cause the reduction in MR with time going on. All patients, especially the young ones, were most likely to benefit from AVR in early time.

**Table:** 

Authors’ roles & responsibilities	
WW	Conceived the study, and participated in its design and coordination and helped to draft the manuscript; final approval of the version to be published
TW	Conceived the study, and participated in its design and coordination and helped to draft the manuscript; final approval of the version to be published
HP	Conceived the study, and participated in its design and coordination and helped to draft the manuscript; final approval of the version to be published
BL	Conceived the study, and participated in its design and coordination and helped to draft the manuscript; final approval of the version to be published
YW	Conceived the study, and participated in its design and coordination and helped to draft the manuscript; final approval of the version to be published
DL	Conceived the study, and participated in its design and coordination and helped to draft the manuscript; final approval of the version to be published
ZZ	Conceived the study, and participated in its design and coordination and helped to draft the manuscript; final approval of the version to be published
RX	Conceived the study, and participated in its design and coordination and helped to draft the manuscript; final approval of the version to be published
KL	Conceived the study, and participated in its design and coordination and helped to draft the manuscript; final approval of the version to be published

## References

[1] Sehovic S, Talic A, Kacila M, Tahirovic E (2015). The influence of aortic valve replacement on functional moderate-to-severe mitral regurgitation in patients with aortic valve stenosis. Acta Inform Med.

[2] Ramani J, Malhotra A, Wadhwa V, Sharma P, Garg P, Tarsaria M (2017). Single-dose lignocaine-based blood cardioplegia in single valve replacement patients. Braz J Cardiovasc Surg.

[3] Matsumura Y, Gillinov AM, Toyono M, Oe H, Yamano T, Takasaki K (2010). Echocardiographic predictors for persistent functional mitral regurgitation after aortic valve replacement in patients with aortic valve stenosis. Am J Cardiol.

[4] Kowalówka AR, Onyszczuk M, Wanha W, Deja MA (2016). Do we have to operate on moderate functional mitral regurgitation during aortic valve replacement for aortic stenosis?. Interact Cardiovasc Thorac Surg.

[5] Singh JP, Evans JC, Levy D, Larson MG, Freed LA, Fuller DL (1999). Prevalence and clinical determinants of mitral, tricuspid, and aortic regurgitation (the Framingham heart study). Am J Cardiol.

[6] Lindman BR, Clavel MA, Mathieu P, Iung B, Lancellotti P, Otto CM (2016). Calcific aortic stenosis. Nat Rev Dis Primers.

[7] Thaden JJ, Nkomo VT, Enriquez-Sarano M (2014). The global burden of aortic stenosis. Prog Cardiovasc Dis.

[8] Khosravi A, Sheykhloo H, Karbasi-Afshar R, Saburi A (2015). Echocardiographic changes after aortic valve replacement: does the failure rate of mitral valve change?. ARYA Atheroscler.

[9] Leavitt BJ, Baribeau YR, DiScipio AW, Ross CS, Quinn RD, Olmstead EM (2009). Outcomes of patients undergoing concomitant aortic and mitral valve surgery in northern new England. Circulation.

[10] Zilberszac R, Gleiss A, Binder T, Laufer G, Grimm M, Gabriel H (2018). Prognostic relevance of mitral and tricuspid regurgitation in patients with severe aortic stenosis. Eur Heart J Cardiovasc Imaging.

[11] Sorabella RA, Olds A, Yerebakan H, Hassan D, Borger MA, Argenziano M (2018). Is isolated aortic valve replacement sufficient to treat concomitant moderate functional mitral regurgitation? A propensity-matched analysis. J Cardiothorac Surg.

[12] Wyler S, Emmert MY, Biaggi P, Seifert B, Grünenfelder J, Falk V (2013). What happens to functional mitral regurgitation after aortic valve replacement for aortic stenosis?. Heart Surg Forum.

[13] Mahmood F, Warraich HJ, Gorman III JH, Gorman RC, Chen TH, Panzica P (2012). Changes in mitral annular geometry after aortic valve replacement: a three-dimensional transesophageal echocardiographic study. J Heart Valve Dis.

[14] Sehovic S, Talic A, Kacila M, Tahirovic E (2015). The influence of aortic valve replacement on functional moderate-to-severe mitral regurgitation in patients with aortic valve stenosis. Acta Inform Med.

[15] Waisbren EC, Stevens LM, Avery EG, Picard MH, Vlahakes GJ, Agnihotri AK (2008). Changes in mitral regurgitation after replacement of the stenotic aortic valve. Ann Thorac Surg.

[16] Malhotra A, Ananthanarayanan C, Wadhawa V, Siddiqui S, Sharma P, Patel K (2018). OPCABG for moderate CIMR in elderly patients: a superior option?. Braz J Cardiovasc Surg.

[17] Isnard R, Acar C (2008). The mitral annulus area: a useful tool for the surgeon. J Heart Valve Dis.

[18] Lim JY, Jung SH, Kim JB, Chung CH, Lee JW, Song H (2014). Management of concomitant mild to moderate functional mitral regurgitation during aortic valve surgery for severe aortic insufficiency. J Thorac Cardiovasc Surg.

